# The Association Between Seeking Urgent Dental Care and the Prevalence of Root Caries Among Patients in the United States, National Health And Nutrition Examination Survey 2017-2020: A Cross-Sectional Study

**DOI:** 10.7759/cureus.41797

**Published:** 2023-07-12

**Authors:** Talha Mobin, Tooba Zahid Khan, Anma Mobin, Muhammad R Tahir, Qirat Imran

**Affiliations:** 1 Dentistry, CMH Lahore Medical College and Institute of Dentistry, Lahore, PAK; 2 Dornsife School of Public Health, Drexel University, Philadelphia, USA; 3 Dentistry, Akhtar Saeed Medical and Dental College, Lahore, PAK

**Keywords:** clinical dentistry, national health and nutrition examination survey (nhanes), prevalence, urgent dental care, root caries

## Abstract

Objective: This cross-sectional study aimed to investigate the association between seeking urgent dental care and the prevalence of root caries among patients in the United States, utilizing data from the Nutritional Health and Examination Survey (NHANES) conducted between 2017 and 2020. Our hypothesis is that patients who are seeking urgent dental care due to pain will have a high prevalence of root caries.

Methods: The study utilized a cross-sectional design, analyzing data from NHANES 2017-March 2020 pre-pandemic data, a nationally representative survey. The sub-sample consisted of 6,186 participants aged 20 years and older who underwent oral health assessments, including the examination of root caries. Information on seeking urgent dental care was obtained through self-reported data. Statistical analyses, using SAS 9.4 (SAS Institute Inc., Cary, NC), were performed to assess the association between urgent care seeking and root caries prevalence while controlling the potential confounding variables. Descriptive statistics and multivariable logistic regression were used.

Results: The study included a total of 6,186 participants. The mean age (SD) of the participants was 49.7 (17.2). Some 52% were females and 48% were males. Some 14% (849) of the samples had root caries and 28% (1739) of the participants were seeking urgent dental care due to pain. Findings indicated a significant association between seeking urgent dental care and the prevalence of root caries (odds ratio, OR = 2.72, 95% confidence interval, CI = 2.32-3.18). Individuals who reported seeking urgent care had a higher prevalence of root caries (26% vs. 9%) compared to those who did not seek urgent care. Socioeconomic factors such as poverty and education, and other factors such as age, gender, race, marital status, and alcohol intake were identified as potential confounders.

Conclusion: This study provides evidence of the association between seeking urgent dental care and the prevalence of root caries among patients in the United States. The findings underscore the importance of promoting regular dental visits, preventive measures, and early interventions to mitigate the risk of root caries among individuals seeking urgent dental care. Addressing socio-economic barriers, improving access to dental services, and enhancing oral health education are crucial steps toward reducing the burden of root caries in this population. Further, longitudinal studies are recommended to establish the temporal relationship between urgent care seeking and root caries development.

## Introduction

Root caries often manifest as a gradually developing defect located on the root of a tooth. This occurs when the tooth's attachment to the surrounding periodontal tissues is compromised to some extent, leading to exposure of the affected root to the oral environment [1-2]. Root caries is any carious lesion that occurs on the root surface of the tooth. Typically, it appears discolored, softened, and poorly defined, affecting both the cementum and the underlying dentin [3]. Root caries lesions are most often located on exposed surfaces. However, some studies have found that 10%-20% of lesions may occur subgingivally [4]. Ravald et al. identified other areas at risk for root caries, including the margin of previous restorations (51%), the cementoenamel junction (CEJ) (25%), and points where lesions merge with other dental issues (17%), particularly in patients undergoing periodontal treatment [5]. Additionally, Katz et al. reported that root caries lesions are commonly found in the following descending order of frequency: buccal and interproximal surfaces of mandibular posterior teeth, interproximal surfaces of anterior maxillary teeth, lingual and interproximal surfaces of maxillary posterior teeth, and buccal and interproximal surfaces of mandibular anterior teeth [6]. The diagnosis and staging of root caries are based on the location, color, texture, cavitation, and contour of the tooth surface involved [7-15]. The diagnosis of root caries primarily relies on conventional visual and tactile examination methods. However, concerns have been raised about the reliability and consistency of this screening approach for root caries [16]. In order to enhance the sensitivity and specificity of the diagnostic process for root caries, radiographs, and existing microbiological tests can be utilized as additional tools to supplement the clinical evaluation [17]. Nonetheless, improving the accuracy of root caries diagnosis still remains a challenge.

In recent years, seeking urgent dental care has emerged as an important consideration in dental research [18]. Individuals who visit dental clinics for urgent dental problems often present with advanced oral health conditions, including severe dental caries [19]. The urgency of these cases may reflect delayed or inadequate preventive care, limited access to dental services, or other socio-economic factors [20]. Understanding the relationship between seeking urgent dental care and the prevalence of root caries could provide valuable insights into the broader oral health landscape and help guide targeted interventions.

To shed light on this relationship, the current study aims to investigate the association between seeking urgent dental care due to pain and the prevalence of root caries among patients in the United States. Our hypothesis is that patients who are seeking urgent dental care due to pain will have a high prevalence of root caries. We utilize data from the Nutritional Health and Examination Survey (NHANES) conducted between 2017 and 2020, a nationally representative survey that includes comprehensive oral health assessments. By analyzing this rich dataset, we can assess the extent to which seeking urgent dental care is linked to root caries prevalence while controlling for relevant confounding factors.

By elucidating the association between seeking urgent dental care and root caries, we can identify high-risk populations and target interventions towards preventive measures, oral health education, and improving access to regular dental care. Ultimately, our aim is to contribute to the development of effective strategies that can reduce the burden of root caries and enhance the overall oral health of the population.

## Materials and methods

Study design and subjects

This is a cross-sectional study using the National Health and Nutrition Examination Survey dataset that was collected during the 2017-March 2020 pre-pandemic period nationwide in the United States. This data was available freely, as open access on the Centers for Disease Control and Prevention's website [21]. The National Health and Nutrition Examination Survey enrolled 0-80 years. For the sub-study, adults, males, and females, 20 years of age and above are used in the sample.

Outcome

The outcome variable was root caries. Root caries were assessed for both, males and females over the ages of 18 years. The oral health examination was carried out by licensed dentists (D.D.S./D.M.D.), serving as dental examiners, who were authorized to practice in at least one U.S. state. To facilitate efficient data collection, a health technician provided support by entering examiner observations directly into a computerized data collection system. These oral health assessments were conducted in a dedicated mobile examination center (MEC) room. The MEC was equipped with essential dental equipment, including a portable dental chair, a light source, and compressed air, ensuring a suitable environment for comprehensive oral examinations. The current survey continued to include a root caries assessment for participants aged 18 years and older. Dental examiners performed a comprehensive "whole mouth" evaluation to identify any untreated root caries and assess the presence of dental root restorations. This standardized approach allowed for a thorough examination of the participants' oral health status, specifically focusing on root caries and related dental treatments. Only the teeth that had recession were examined for root caries. Each quadrant with recession was dried with air and examined with a surface-reflecting mirror and a No. 23 explorer. All participants with root caries were coded as “1” if they were diagnosed with it and as “0” if they did not have root caries. All participants with missing data with regard to the outcome were excluded.

Exposure

The exposure variables were “seeking urgent dental care.” This was a question in the survey which asked all the participants “What was the main reason for your last dental visit?” We categorized the answers to this question into “seeking urgent dental care” if the participants stated that something was wrong, bothering them or hurting them due to which they went to see a dentist. The participants were coded as “1” if they went to see the dentist due to pain and coded as “0” otherwise. All participants with missing data were excluded from the final sample.

Confounders

Confounders were identified a priori by consulting relevant literature [22-23]. We used diagrams to think about the causal process underlying our hypothesis. We used Daggity software (Tumor Immunology Lab and Institute for Computing and Information Sciences, Radboud University, Nijmegen, Netherlands) to help us identify variables that should not be included as confounders (decedents of the exposure [mediators] or descendants of the outcome or colliders). Through this process, we identified at last one minimal adjustment set required to control for confounding and worked to ensure that a minimal set was in the analyses. 

To control for confounding in the association between exposure and outcome, the variables age, gender, race/ethnicity, smoking, drinking alcohol, education, socioeconomic status, and marital status were used and adjusted for, which furthermore indicates our minimal adjustment set.

Older individuals are more likely to experience root caries due to factors like gum recession, decreased saliva production, and longer exposure to risk factors. At the same time, older individuals may have a higher tendency to seek urgent dental care due to various age-related factors. Thus, age can confound the association between root caries and seeking urgent dental care [24]. There might be differences in oral health behaviors and attitudes between genders. For example, women may have better oral hygiene practices compared to men. Additionally, hormonal factors and societal norms can influence dental care-seeking behavior. Therefore, gender can be a confounding variable in the association between root caries and seeking urgent dental care [25]. Oral health disparities exist among different racial and ethnic groups, often influenced by socioeconomic factors, access to care, cultural practices, and genetic factors. These disparities can affect both the prevalence of root caries and the likelihood of seeking urgent dental care, making race a potential confounder in the association [26]. Socioeconomic factors, such as income, and education can influence both the development of root caries and the ability to access dental care. Individuals with lower socio-economic status (SES) may have limited resources, inadequate insurance coverage, and barriers to seeking timely dental care, while higher SES individuals may have better access and resources. Thus, SES acts as a confounding variable in the relationship between root caries and seeking urgent dental care [27]. Smoking is a known risk factor for poor oral health, including root caries. Smokers are more likely to have compromised immune systems, reduced saliva flow, and increased plaque accumulation, contributing to the development of root caries. Additionally, smoking may affect oral health-seeking behavior, as smokers may have a higher threshold for seeking dental care. Therefore, smoking can confound the association between root caries and seeking urgent dental care [28]. Excessive alcohol consumption can negatively impact oral health, including an increased risk of root caries. Alcohol abuse can lead to dehydration, poor oral hygiene, and impaired healing processes. Moreover, alcohol misuse may affect an individual's priorities and willingness to seek dental care promptly. Hence, alcohol use can act as a confounder in the relationship between root caries and seeking urgent dental care [29]. Marital status can influence oral health in various ways. For instance, married individuals may have better social support, leading to improved oral health behaviors. They might also have more financial resources or access to dental insurance through a spouse's plan. These factors can affect both the occurrence of root caries and the likelihood of seeking urgent dental care, making marital status a confounding variable [30]. Education level can influence oral health knowledge, awareness, and behaviors. Individuals with higher education may be more knowledgeable about preventive measures, have better oral hygiene practices, and seek dental care more frequently. On the other hand, lower education levels might lead to limited oral health literacy and delayed care-seeking. Hence, education can confound the association between root caries and seeking urgent dental care [31].

Analytic sample and missing data

Among the 15,560 participants who contributed to the data, 6,328 participants were excluded for not having any data related to our research. Further exclusions were made as follows: 171 without an exposure measurement and 1,633 without an outcome measurement and 2,659 who did not have full covariate data. The total missing data from the subset is 3,046 participants (33% of the subset). Following the complete-case analysis approach, the final analytic sample was 6,186 participants (40% of the original sample). Sample characteristics for included vs. excluded are shown in the table in the Appendix. On average, the excluded sample was similar to those who were included in most characteristics.

 Statistical method

The statistical package used for the analysis was SAS 9.4 (SAS Institute Inc., Cary, NC). Unadjusted descriptive statistics (percentages and means) examined participants' characteristics relative to the exposure (seeking urgent dental care) and outcome (root caries). Multivariable adjusted logistic regression was used to model the relative odds of having root caries. Confounders were selected a priori based on our literature review and directed acyclic graphs (see Methods section). Model 1 was the unadjusted model. Model 2 adjusted for age and gender. Model 3 added race and education to age and gender, and Model 4 added socioeconomic status, alcohol, and marital status. A p-value of 0.05 and 95% CI was used to test for statistically significant associations.

## Results

Descriptive results

Demographics and other characteristics of the participants are reported in Table 1. The total number of participants who were included in the analysis was 6,186. Among these 52% (N=3194) were females and 48% (N=2992) were males. The mean age of the participants was 49.7 years. Some 28% (N=1739) of participants sought urgent dental care due to pain and 14% (N=849) had root caries. The majority of the participants (37%) were Non-Hispanic White and 26% were Non-Hispanic Black. Some 61% of participants had a greater than high school education, 23% had a high school education, and 15% had less than a high school education. Some 18% of participants were poor [had a poverty income ratio (PIR) less than 1], 26% were near poor, and 57% were not poor. Some 59% of participants were married, 21% were divorced, and 20% had never married. Some 92% of participants had alcohol at least once in their life and 8% had never had alcohol. 

**Table 1:** Descriptive characteristics of total participants (N=6,186). N, number of participants; SD, standard deviation

Characteristics	N	%
Mean age (SD)	49.7 (17.2)	-
Gender	-	
Male	2992	48%
Female	3194	52%
Race/Ethnicity	-	
Multi-race	308	5%
White	2275	37%
Black	1599	26%
Mexican	700	11%
Other	606	10%
Asian	698	11%
Education	-	
Less than High School	943	15%
High School	1446	23%
Greater than High School	3794	61%
Marital Status	-	
Married	3653	59%
Divorced	1312	21%
Never Married	1219	20%
Poverty	-	
Poor	1084	18%
Near poor	1602	26%
Not poor	3500	57%
Alcohol	-	
Has had at least one drink	5675	92%
Never drank	511	8%

There were 849 (14%) participants who had root caries and 5337 (86%) did not have root caries. There were 1739 (28%) participants who sought urgent dental care and 4447 (72%) were not seeking urgent dental care. These results are shown in Table 2.

**Table 2:** Distribution of participants who sought urgent dental care and root caries.

	Yes (%)	No (%)	Total (%)
Root caries	849 (14%)	5337 (86%)	6186 (100%)
Seeking urgent dental care	1739 (28%)	4447 (72%)	

Among those who had root caries, 54% (N=457) were seeking urgent dental care due to pain. The mean age of these participants was 54.1 years; 56% (N=474) were males and 44% (N=375) were females. Some 36% of participants were Non-Hispanic Whites and 37% were Non-Hispanic Blacks. Among participants who had root caries, 46% had a greater than high school education, 30% had a high school education, and 23% had less than a high school education. Some 30% of participants had a PIR of less than 1, 38% were near poor, and 32% were not poor. Some 48% of the participants who had root caries were married, 31% were divorced, and 20% were never married. Some 92% of participants with root caries had alcohol at least once. Among those participants who were seeking urgent dental care (N=1739), 26% had root caries and 74% did not have root caries. The mean age of these participants who were seeking urgent dental care was 51.1 years. Some 52% of these participants were males and 48% were females. Some 35% of these participants were Non-Hispanic Whites and 32% were Non-Hispanic Blacks. Among participants who were seeking urgent dental care, 46% had a greater than high school education, 31% had a high school education, and 22% had less than a high school education. Some 39% of these participants were not poor, 34% were near poor, and 27% were poor. Some 55% of the participants were married, 25% were divorced, and 20% were never married. Among participants who were seeking urgent dental care, 92% have had alcohol at least once in their life. These results are shown in Table 3.

**Table 3:** Distribution of sample characteristics for total participants (N=6186), stratified by root caries and seeking urgent dental care. N, number of participants; SD, standard deviation

Characteristics (N=6,186)	Root caries (N, Column %)		Seeking urgent dental care (N, Column %)	
	Yes (N=849, 14%)	No (N=5337, 86%)	Yes (N=1739, 28%)	No (N=4447, 72%)
Mean Age (SD)	54 (15.8)	49 (17.4)	51.1 (16.3)	49.1 (17.6)
Gender	-			
Male	474 (56%)	2518 (47%)	905 (52%)	2087 (47%)
Female	375 (44%)	2819 (53%)	834 (48%)	2360 (53%)
Race/Ethnicity	-			
Multi-race	53 (6%)	255 (5%)	92 (5%)	216 (5%)
White	309 (36%)	1966 (37%)	608 (35%)	1667 (37%)
Black	317 (37%)	1282 (24%)	565 (32%)	1034 (23%)
Mexican	71 (8%)	629 (12%)	204 (12%)	496 (11%)
Other	47 (6%)	559 (10%)	158 (9%)	448 (10%)
Asian	52 (6%)	646 (12%)	112 (6%)	586 (13%)
Education	-			
Less than High School	197 (23%)	746 (14%)	389 (22%)	554 (13%)
High School	258 (30%)	1188 (22%)	545 (31%)	901 (20%)
Greater than High School	393 (46%)	3401 (64%)	805 (46%)	2989 (67%)
Marital Status	-			
Married	411 (48%)	3242 (60%)	950 (55%)	2703 (60%)
Divorced	265 (32%)	1047 (20%)	437 (25%)	875 (20%)
Never married	173 (20%)	1046 (20%)	352 (20%)	867 (20%)
Poverty	-			
Poor	251 (30%)	833 (16%)	470 (27%)	614 (14%)
Near poor	323 (38%)	1279 (24%)	591 (34%)	1011 (23%)
Not poor	275 (32%)	3225 (60%)	678 (39%)	2822 (63%)
Alcohol	-			
Has had at least one drink	69 (8%)	442 (8%)	133 (8%)	378 (8%)
Never drank	780 (92%)	4895 (92%)	1606 (92%)	4069 (92%)

Primary adjusted analysis

Table 4 displays the progressive adjustment for the covariates in each of the three models. Results of the final model indicate a positive association between seeking urgent dental care and root caries. That is, after controlling for all confounders such as age, gender, race, education, marital status, poverty, and alcohol, the odds of having root caries are 2.72 times the odds in people seeking urgent dental care due to pain compared to people not seeking urgent dental care (OR = 2.72, 95% CI = 2.32-3.18). A strong positive association was found between root caries and seeking urgent dental care alone (OR = 3.69, 95% CI = 3.18-4.28). After adjusting for age and gender, root caries and seeking urgent dental care had a somewhat weaker association (OR = 3.59, 95% CI = 3.09-4.18) and after adjusting for age gender, race, and education, root caries and seeking urgent dental care had a weaker association (OR = 3.09, 95% CI = 2.65-3.61).

**Table 4:** Adjusted OR and 95% CI of the association between root caries and seeking urgent dental care. *Statistically significant result (p-value < 0.05) OR, odds ratio; CI, confidence interval

Root caries = yes				
-		95% CI		
-	OR	Low	High	p-Value
Model 1: Unadjusted Model	3.69	3.18	4.28	<0.0001*
Model 2: Adjusted for Age and Gender	3.59	3.09	4.18	<0.0001*
Model 3: Adjusted for Age, Gender, Race, and Education	3.09	2.65	3.61	<0.0001*
Model 4: Adjusted for Age, Gender, Race, Education, Socioeconomic status, Marital Status, and Alcohol	2.68	2.29	3.14	<0.0001*

## Discussion

The present study aimed to investigate the association between seeking urgent dental care and the prevalence of root caries among patients in the United States. By analyzing data from the National Health and Examination Survey (NHANES) conducted between 2017 and 2020, we sought to identify potential links between urgent dental care utilization and the occurrence of root caries while considering relevant confounding factors. Our findings revealed a significant association between seeking urgent dental care and the prevalence of root caries (p < 0.0001). Patients who reported seeking urgent dental care exhibited a higher prevalence of root caries compared to those who did not seek urgent care. This suggests that individuals who seek urgent dental care may be at a greater risk of developing root caries, indicating a need for targeted preventive interventions and improved access to regular dental services. Furthermore, the odds of root caries in males who were seeking urgent dental care were 26% higher than in females. This was a statistically significant association (p < 0.0001). A study by Su et al. confirmed that males were more likely to visit the dentist seeking urgent care due to pain and hence have more advanced dental decay including root caries [32].

A study conducted by Du et al. examined the oral health status of individuals seeking urgent dental care in the United States [33]. The researchers found that patients presenting with urgent dental conditions had a higher prevalence of untreated root caries compared to individuals seeking routine dental care. This aligns with our findings, suggesting that delayed or inadequate dental care among those seeking urgent treatment may contribute to the higher incidence of root caries.

Another study by Mamai-Homata et al. explored the association between risk indicators and the occurrence of root caries among older adults [34]. The results indicated that individuals who primarily sought dental care for urgent needs had a significantly higher risk of developing root caries. These findings corroborate our study's findings and emphasize the importance of regular dental visits in preventing root caries.

In a population-based study conducted by Hariyani et al., the researchers investigated the impact of socioeconomic factors on dental care utilization and oral health outcomes [35]. The study found that individuals from lower socio-economic backgrounds were more likely to seek urgent dental care due to financial constraints and limited access to routine dental services. This aligns with our discussion of the potential role of socio-economic factors and limited access to dental care in the association between urgent care seeking and root caries prevalence.

Furthermore, a systematic review by Hayes et al. examined the risk indicators associated with root caries [26]. The review highlighted the importance of oral health education, regular dental visits, and early interventions to prevent root caries. Implementing targeted oral health education programs and improving access to preventive dental care aligns with the recommendations proposed in our Discussion section.

Moreover, in our study, the participants who reported having an education greater than high school had a decrease in the odds of root caries by 45% compared to participants who had less than a high school education. A study done by Islas-Granillo et al. found that people who had higher education were more likely to utilize dental care services and hence, had fewer oral health problems, including root caries [36].

A study by Sen et al. found that individuals who reported seeking urgent dental care had a higher likelihood of having untreated root caries compared to those who sought regular dental care [37]. This observation supports the notion that delayed or inadequate dental care can contribute to the occurrence of root caries. Furthermore, Sen et al. emphasized the importance of early preventive interventions and regular dental visits to prevent the progression of dental caries and reduce the burden of root caries among high-risk populations.

The strengths of this study are that the study utilized data from the Nutritional Health and Examination Survey (NHANES), which is a nationally representative survey. This ensures that the findings can be generalized to the larger population in the United States. The NHANES survey includes comprehensive oral health assessments conducted by licensed dentists, ensuring standardized and reliable measurements of root caries prevalence. The use of trained dental examiners enhances the validity and accuracy of the collected data. The study analyzed data from the NHANES survey conducted between 2017 and 2020. The extended duration allows for a more robust examination of the association between seeking urgent dental care and the prevalence of root caries, potentially capturing variations over time. The study accounted for relevant confounding variables such as age, gender, SES, race, education, marital status, and alcohol. By controlling for these factors, the study aimed to isolate the specific association between seeking urgent dental care and root caries prevalence. The study's findings have direct implications for oral health professionals and policymakers. By identifying the association between urgent care seeking and root caries prevalence, the study highlights the need for preventive strategies, oral health education, and improved access to regular dental care, potentially leading to improved oral health outcomes for patients.

It is important to acknowledge the limitations of our study. The cross-sectional nature of the NHANES data limits our ability to establish causality or determine the temporal relationship between urgent dental care seeking and root caries development. Longitudinal studies with a prospective design would provide stronger evidence for understanding the impact of urgent dental care utilization on root caries prevalence. Furthermore, sampling bias was an issue in our study since only 30% of our sample was 40 years of age or below. The over-40 age group has a higher prevalence of root caries than the under-40 group. The issue led to an increased number of observations with the outcome but did not have an effect on the relationship between it and the exposure, leading to the conclusion that it was sampling bias, as it was only related to the outcome. Furthermore, residual confounding might have been present. Sugar intake might have confounded the relationship between seeking urgent dental care and root caries. Since sugar intake has a positive association with root caries and urgent dental care, the measure of association would be biased away from the null. Furthermore, some participants might have had non-odontogenic orofacial pain, due to which they would have needed urgent dental care. This might have added a non-differential misclassification error of the exposure (seeking urgent dental care due to pain) hence, biasing the association towards the null. The study relied on self-reported data for variables such as seeking urgent dental care. Self-reporting is subject to recall bias and may be influenced by social desirability bias, potentially impacting the accuracy of the reported information. Objective measures of dental care utilization would enhance the reliability of the findings.

## Conclusions

The present study investigated the association between seeking urgent dental care and the prevalence of root caries among patients in the United States using data from the NHANES survey conducted between 2017 and 2020. The results of our analysis revealed a significant association between root caries and the likelihood of seeking urgent dental care, even after controlling for confounders. Our study's findings suggest that individuals with root caries are more likely to seek urgent dental care compared to those without this condition. This association highlights the importance of prompt dental care-seeking behavior in individuals affected by root caries, as it may be indicative of their recognition of the severity and urgency of the condition. By identifying this significant relationship, our study contributes to the existing body of knowledge regarding the impact of root caries on healthcare-seeking behaviors. These findings may have important implications for oral health professionals, policymakers, and public health organizations in terms of developing targeted interventions and strategies aimed at promoting early detection and timely management of root caries. Further research is warranted to investigate the underlying mechanisms and factors driving the association between root caries and urgent dental care-seeking behavior.

## Appendices

**Table 5:** Sample characteristics for total and included vs. excluded participants, showing patterns in missingness. SD, standard deviation

Characteristic		Total with missing (%)	Included (%)	Excluded (%)
Root Caries	No	6520 (71%)	5337 (86%)	1183 (84%)
	Yes	1079 (12%)	849 (14%)	230 (16%)
	Missing	1633 (18%)		1633
Seeking Urgent Dental Care	No	6398 (69%)	4447 (72%)	1951 (68%)
	Yes	2663 (29%)	1739 (28%)	924 (32%)
	Missing	171 (2%)		171
Age	Mean (SD)	51.1 (17.7)	49.7 (17.2)	54.1 (18.2)
Gender	Male	4479 (49%)	2992 (48%)	1487 (49%)
	Female	4753 (51%)	3194 (52%)	1559 (51%)
	Missing	0 (0%)		0
Race/Ethnicity	Multi-race	439 (5%)	308 (5%)	131 (4%)
	White	3217 (35%)	2275 (37%)	942 (31%)
	Black	2459 (27%)	1599 (26%)	860 (28%)
	Mexican	1057 (11%)	700 (11%)	357 (12%)
	Other	940 (10%)	606 (10%)	334 (11%)
	Asian	1120 (12%)	698 (11%)	422 (14%)
	Missing	0 (0%)		0
Marital Status	Married	5279 (57%)	3653 (59%)	1626 (54%)
	Divorced	2148 (23%)	1312 (21%)	836 (28%)
	Never married	1795 (19%)	1219 (20%)	576 (19%)
	Missing	0 (0%)		0
Education	Less than High School	1760 (19%)	943 (15%)	817 (27%)
	High School	2225 (24%)	1446 (23%)	779 (26%)
	Greater than High School	5232 (57%)	3794 (61%)	1438 (47%)
	Missing	0 (0%)		0
Poverty	Poor	1496 (16%)	1084 (18%)	412 (25%)
	Near poor	2114 (23%)	1602 (26%)	512 (31%)
	Not poor	4218 (46%)	3500 (57%)	718 (44%)
	Missing	1404 (15%)		1404
Alcohol	Never drank	742 (8%)	511 (8%)	231 (13%)
	Drank at least once	7235 (78%)	5675 (92%)	1560 (87%)
	Missing	1255 (14%)		1255
